# Leveraging big data for public health: Mapping malaria vector suitability in Malawi with Google Earth Engine

**DOI:** 10.1371/journal.pone.0235697

**Published:** 2020-08-04

**Authors:** April N. Frake, Brad G. Peter, Edward D. Walker, Joseph P. Messina

**Affiliations:** 1 Department of Geography, The University of Alabama, Tuscaloosa, Alabama, United States of America; 2 Department of Microbiology and Molecular Genetics, Michigan State University, East Lansing, Michigan, United States of America; 3 College of Arts and Sciences, The University of Alabama, Tuscaloosa, Alabama, United States of America; Montana State University, UNITED STATES

## Abstract

In an era of big data, the availability of satellite-derived global climate, terrain, and land cover imagery presents an opportunity for modeling the suitability of malaria disease vectors at fine spatial resolutions, across temporal scales, and over vast geographic extents. Leveraging cloud-based geospatial analytical tools, we present an environmental suitability model that considers water resources, flow accumulation areas, precipitation, temperature, vegetation, and land cover. In contrast to predictive models generated using spatially and temporally discontinuous mosquito presence information, this model provides continuous fine-spatial resolution information on the biophysical drivers of suitability. For the purposes of this study the model is parameterized for *Anopheles gambiae* s.s. in Malawi for the rainy (December–March) and dry seasons (April–November) in 2017; however, the model may be repurposed to accommodate different mosquito species, temporal periods, or geographical boundaries. Final products elucidate the drivers and potential habitat of *Anopheles gambiae* s.s. Rainy season results are presented by quartile of precipitation; Quartile four (Q4) identifies areas most likely to become inundated and shows 7.25% of Malawi exhibits suitable water conditions (water only) for *Anopheles gambiae* s.s., approximately 16% for water plus another factor, and 8.60% is maximally suitable, meeting suitability thresholds for water presence, terrain characteristics, and climatic conditions. Nearly 21% of Malawi is suitable for breeding based on land characteristics alone and 28.24% is suitable according to climate and land characteristics. Only 6.14% of the total land area is suboptimal. Dry season results show 25.07% of the total land area is suboptimal or unsuitable. Approximately 42% of Malawi is suitable based on land characteristics alone during the dry season, and 13.11% is suitable based on land plus another factor. Less than 2% meets suitability criteria for climate, water, and land criteria. Findings illustrate environmental drivers of suitability for malaria vectors, providing an opportunity for a more comprehensive approach to malaria control that includes not only modeled species distributions, but also the underlying drivers of suitability for a more effective approach to environmental management.

## Introduction

Despite a long, complicated history of disease control efforts, malaria remains a significant challenge to global public health [1]. Human malaria is caused by a single-celled parasite (*Plasmodium*) transmitted by mosquitoes of the *Anopheles* genus [2, 3]; approximately seventy *Anopheles* species vector malaria parasites [4, 5]. Malaria is a disease of poverty with poorer countries disproportionately bearing the largest burden of infection [6, 7]. Prevention and control encompasses numerous techniques including targeting either vectors or parasites [8, 9] making malaria vector distribution maps an integral component of vector control strategies [10].

The literature on predicted distributions of malaria vector species’ is robust (e.g., [5, 10–13]). An important distinction between different types of models is their intended aim: modeling species fundamental or realized niche. A fundamental niche refers to the geographic space wherein environmental conditions permit the species to exist, absent of biotic interactions; realized niche is the space the species is known to occupy, including biotic interactions such as competitive exclusion [14–16]. As phrased by Guisan & Zimmerman [16], “*Differentiating between the fundamental and the realized niche of a species is particularly important because it distinguishes whether a simulated distribution is predicted from theoretical physiological constraints or rather from field-derived observations*.” Modeling fundamental niche can be expressed as a combination of environmental variables that meet species’ physiological and habitat requirements. Conventionally, realized niche has been modeled using methods such as Species Distribution Models (SDM) that depend on presence and/or absence data that reflect the species distribution observed empirically [17, 18]. Such methods allow projections of where species might expand in range to suitable habitat outside the observed or “native” range, as in the case of invasive species.

A notable open-access repository of malariometric data, including maps of predicted vector distributions is the Malaria Atlas Project (MAP). The MAP is a non-profit collaborative of researchers working to “*disseminate free*, *accurate*, *and up to date geographical information on malaria and associated topics*” [19]. MAP includes global databases on malaria risk, vector occurrence, modeled species distributions, cross-sectional surveys of parasite rate, and environmental data pertinent to malaria transmission. The MAP includes five types of vector occurrence data, categorized as: Multiple Vectors (dominant and secondary), Vector Occurrence (Archive), Vector Occurrence (Current), and Vector Occurrence Surveys. Data from MAP are regularly cited in prominent, international research studies (e.g., [20–22]) including the World Health Organization’s World Malaria Report [23].

Dominant species distributions data for MAP are taken from Sinka et al.'s [5] global map of dominant malaria vectors produced using Boosted Regression Tree (BRT) methodology for forty-one dominant vector species. Input data include environmental and climatic grids at a spatial resolution of 5-km [24], presence and/or absence of species data acquired through comprehensive literature searches conducted by Hay et al. [10], and expert opinions on species geographic range [5]. Forty-one predictive distribution maps at a 5-km spatial resolution were generated using BRT, then combined to indicate species presence with a >0.5 probability of occurrence [5]. Where dominance of one species in an area is unknown, two or more species were grouped [5]; for example, for the dominant sub-Saharan African malaria vectors, there were four combinations of species presence data: (1) *Anopheles gambiae* s.s., *Anopheles arabiensis* and *Anopheles funestus* (80.86%), (2) *An*. *arabiensis* and *An*. *funestus* (15.16), (3) *An*. *gambiae* s.s and *An*. *funestus* (2.4%), and (4) *An*. *funestus* (1.58%).

The archived predicted distribution maps for MAP were produced by Sinka et al. [25]. Predictions were generated using similar methodology to the dominant species distributions maps using BRT models (see: [5]). Predicted distribution data are presented for the sub-Saharan Africa region at a 5-km spatial resolution, on a scale of 0–1. Current predicted distributions of malaria vectors for MAP are produced by Wiebe et al., [13] at ~5km resolution. Models were constructed using BRT methodology where presence data included previously collected species data culminated from a combination of literature review and insecticide resistance records. Results elucidate a relative probability of occurrence not only for *An*. *gambiae*, but sibling species within the *Anopheles gambiae* s.l. species complex.

A limitation of existing modeled products of malaria vector species distributions is that their output spatial and temporal resolutions inadequately characterize local scale vector habitat or actual presence. Moreover, many SDMs rely on sampled presence data that are spatially discontinuous and temporally static. Further, there is a gap in information related to the drivers of environmental suitability that work to define vector species’ distributions. It is one thing to predict where a species might exist in space; it is quite another to address what conditions allow or prohibit a species from occupying an area in a given timeframe (see: [26]). Information on the drivers of physiological suitability (or unsuitability) can have considerable implications for vector management as countries continue to work toward malaria control in the wake of changing environmental conditions. In resource-poor settings unable to conduct regular surveys, the availability of high-precision remote sensing information on potential drivers and distributions of vectors can fill a critical deficiency of data and complement existing MAP data products.

The availability of satellite-derived global climate, terrain, and land cover data is transforming the ability to model the suitability of disease vectors across geographies and time scales, and at fine spatial resolutions. Leveraging Google Earth Engine, a raster-based mosquito suitability model was constructed using data as fine as 30-m spatial resolution. The model’s outcomes are intended to assist in identifying *Anopheles gambiae* s.s. fundamental niche and plausible mosquito habitat in Malawi to assist with subsequent spatially targeted management efforts for malaria mitigation. Additionally, any outcome must incorporate seasonality as a primary influence on the fundamental niche owing to the interaction of precipitation, human activity such as cultivation and irrigation for agriculture, and the resultant appearance and disappearance of larval habitats [12, 27]. For the purposes of this study the model is parameterized for *Anopheles gambiae* s.s. in Malawi for the rainy (December–March) and dry seasons (April–November) in 2017 [28, 29]; however, the model may be re-parameterized for any mosquito species (where adequate characterization of the species physiological requirements for survival are available), time periods, or geographical boundaries. For Malawi, it is particularly applicable to *Anopheles arabiensis*, a sympatric member of the *Anopheles gambiae* s.s. species complex and a dominant vector in much of the country [30].

### Google Earth Engine

Google Earth Engine (GEE) is an open-access, cloud-based platform designed for users to ingest and process either their own private data, or work with data from GEE’s multi-petabyte geospatial catalog [31]; newly acquired global monitoring satellite image data from platforms such as NASA MODIS (Moderate Resolution Imaging Spectroradiometer) are added to the GEE repository as they become available. Users access GEE through a web programming interface with an extensive collection of geospatial analytical tools. While traditional methods of data storage and analysis typically preclude users from storing, managing, and processing very large geospatial datasets, GEE removes these barriers, allowing users to more readily process data and disseminate their results [31]. For a complete review of the GEE platform, including system architecture and data distribution models see [31]. The GEE platform offers a generalizable model across spatial and temporal scales, enhances accessibility, and ensures that the widest possible audience may access and manipulate the model without limitations related to data storage or computational processing power.

### Malawi and *Anopheles gambiae* s.s.

**Fig 1:**
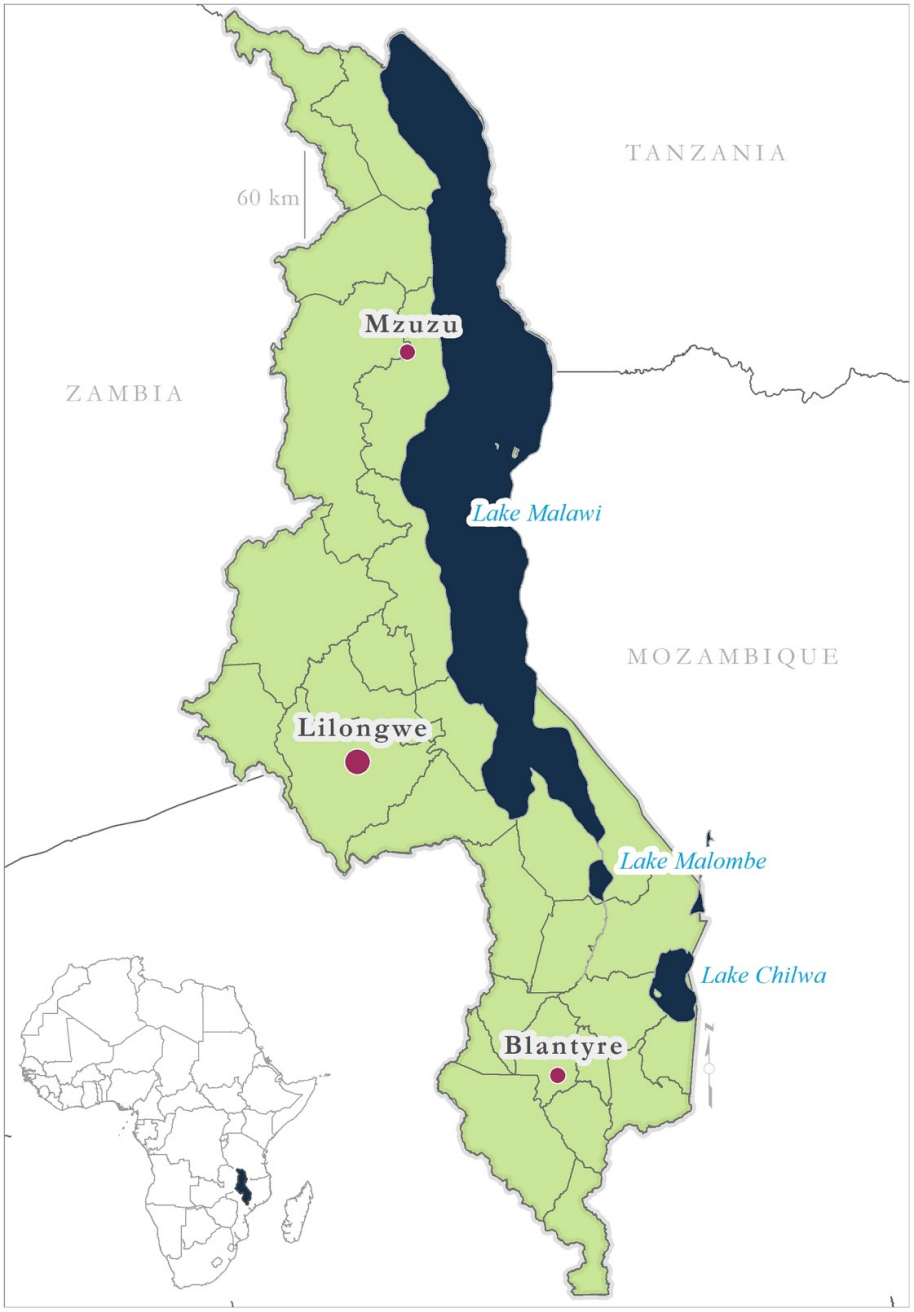
Malawi’s location in sub-Saharan Africa and its major water bodies. Major cities and lakes highlighted.

Malawi is a landlocked country in southern Africa with a predominantly agrarian society [32] and long-standing burden of malaria (Fig 1). In 2017, the World Health Organization reported 4,901,344 confirmed cases of malaria and 3,614 deaths as a result of the disease [33]. Malaria is endemic and stable throughout Malawi, albeit with marked variations in spatio-temporal response to seasonal climate patterns. Areas of highest infection risk are concentrated along the Lake Malawi lakeshore, within the Shire River valley, and the central plains [34, 35]. These areas are characteristically hotter and wetter than other parts of the country. *Plasmodium falciparum*, the most harmful of malaria parasites [36], accounts for 100% of infection in Malawi [37]. Dominant vectors of transmission in Malawi are *An*. *arabiensis*, *An*. *gambiae* s.s., *and An*. *funestus* [37]. *Anopheles gambiae* s.s. is an efficient vector in transmitting malaria [35] and belongs to the *Anopheles gambiae* sensu lato (s.l.) species complex, itself comprised of nine morphologically indistinguishable sibling species: *An*. *gambiae* sensu stricto (s.s.), *An*. *coluzzii*, *An*. *arabiensis*, *Anopheles quadriannulatus* species A, *Anopheles quadriannulatus* species B, *Anopheles melas*, *Anopheles merus*, *Anopheles amharicus*, and *Anopheles bwambae* [38, 39]. The geographical distribution of *An*. *gambiae* s.s. is widespread across sub-Saharan Africa, though predominately concentrated along 10°N latitude and between 10–20°S latitude in Tanzania, Malawi, Mozambique, and Zambia [13]. *Anopheles coluzzii* was elevated to species status from the so-called molecular M biotype whilst *An*. *gambiae* s.s. was synonymized with the molecular S form. *An*. *gambiae* s.s. is modeled here as a single species, rather than these molecular forms or the taxa to which they refer.

## Materials and methods

### Model construction

**Fig 2:**
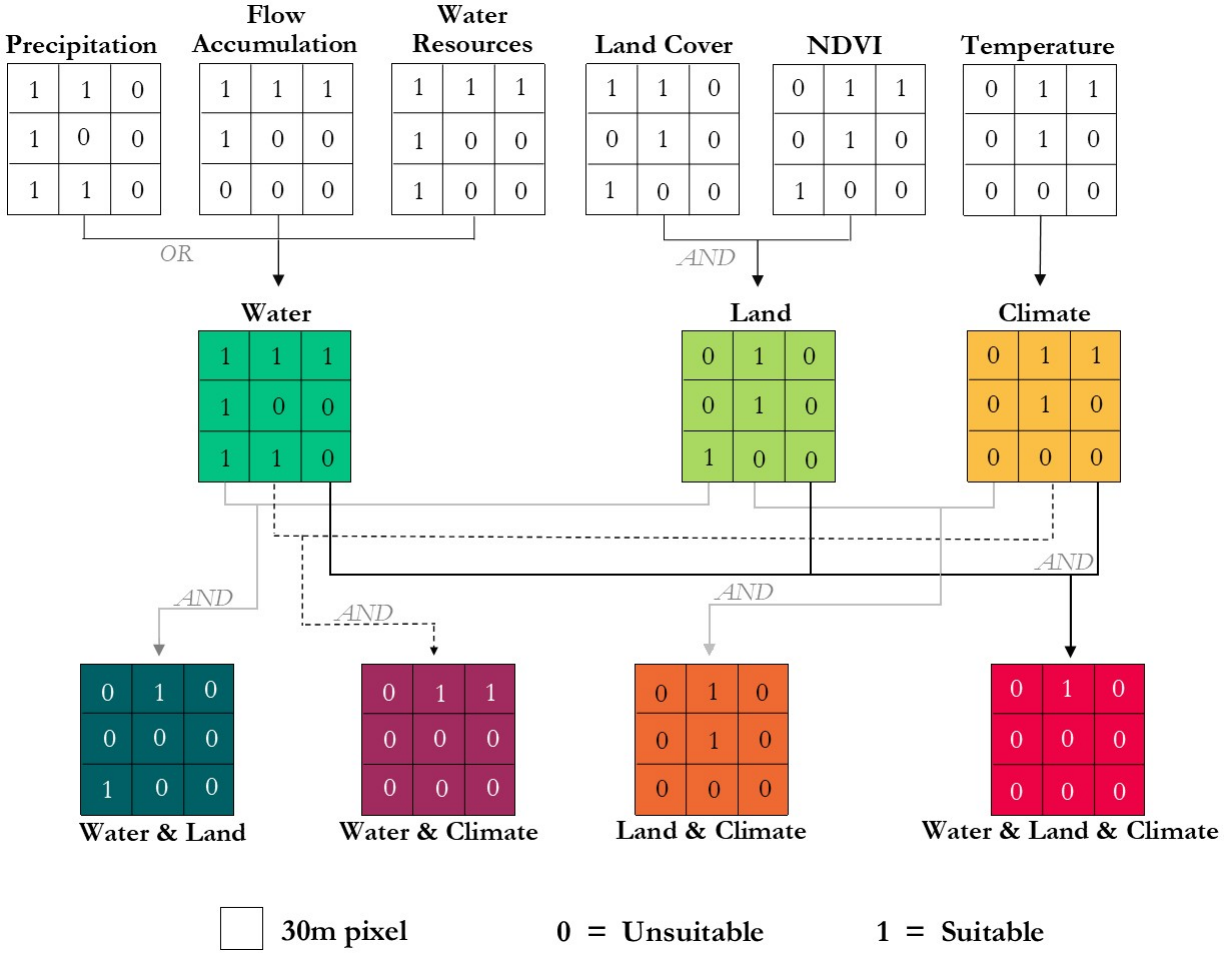
Model conceptual framework. Suitability is defined by the parameter thresholds for each input variable—refer to Table 1.

The spatio-temporal distribution of *An*. *gambiae* s.s. was determined using a raster-based environmental suitability model constructed in GEE that uses abiotic and biotic (i.e., living organism) variables specific to the species’ biological requirements and habitat preference. Suitable areas are defined as those that facilitate the creation and persistence of breeding sites for oviposition and development of *An*. *gambiae* s.s. larvae. Parameter thresholds for each of the input variables were selected based on published findings in the scientific literature (see below) regarding *An*. *gambiae* s.s. habitat: Temperature, Normalized Difference Vegetation Index (NDVI), Land Cover, Precipitation, Flow Accumulation, and Water Resources (Table 1). Each threshold was used to create binary, (suitable [1] or unsuitable [0]), maps for each predictor during the defined time-period. All predictor variable maps are then combined using Boolean logic to produce suitability maps for the target species. Results are displayed by combined suitability types (Climate, Land, Water) to convey the factors contributing to suitability. Temperature was the sole climate variable; NDVI and Land Cover were combined using an ‘AND’ operator to create the binary land suitability grid; Precipitation, Flow Accumulation, and Water Resources were combined using an ‘OR’ operator to create the binary water suitability grid (Fig 2). The ‘OR’ operator was selected for the water suitability grid to ensure that permanent water bodies, dambos, and small depressions were all captured.

**Table 1:** Model parameterization and data sources used for habitat characterization.

	Data Source	Product	Spatial Resolution	Temporal Resolution	Threshold
Temperature	NASA MODIS	MOD11A2.006	1-km	8-day	min: 18°C max: 32°C
NDVI	NASA MODIS	MOD13Q1.006	250-m	16-day	min: 0.35
Land Cover	ESA & UCLouvain	GlobCover 2009 V2.3	300-m	-	see Table 2
Precipitation	UCSB Climate Hazards Group	CHIRPS Pentad	~5.5-km	Pentad	min: 330mm max: 3224mm Q1–4*
Flow Accumulation	WWF	HydroSHEDS	500-m	-	>Q2**
Water Resources	JRC	GSW1_0	30-m	-	>0% water occurrence

*Quartiles 1–4 of the precipitation range are presented to identify areas of varying water inundation potential.

**A threshold for flow accumulation areas and streams was selected using visual imagery interpretation.

### Suitability data and parameters

Suitability parameters are partitioned into three categories: climate, land, and water. Data sources, resolutions, and threshold values are presented in Table 1.

### Climate

#### Temperature

Temperature is critical to mosquito life-history [40–42]. During aquatic life stages, higher ambient temperatures encourage faster development, but have also been shown to cause declining larval survivorship. Likewise, higher environmental temperature increases adult mortality [43]. Bayoh & Lindsay [44] demonstrated that the lower and upper temperature thresholds for *An*. *gambiae* s.s. larval development were 18°C and 32°C. At higher (38–40°C) or lower (10–12°C) temperatures, larval survivorship was reduced and development hindered. Temperature of water in occupied larval habitats near the equator in western Kenya never exceeded 35° C, whereas nearby mud and soil reached as high as 50° C [45]. Throughout most of sub-Saharan Africa, larvae may regularly experience high temperatures, particularly during dry seasons, though in some cases only for a limited number of hours during the day. Because eggs are commonly found on moist soil around larval habitats [45, 46] and temperatures of that soil commonly exceed 40° C, high temperature is a limiting factor to distribution.

Minimum and maximum temperature thresholds in this model were set to 18°C and 32°C, respectively [44]. Data were acquired from the Moderate Resolution Imaging Spectroradiometer (MODIS) Terra Land Surface Temperature and Emissivity 8-Day Global dataset (MOD11A2 V006) at a 1-km spatial resolution [47] Data are available from 02-18-2000 through the present (correct as of 01-01-2020).

### Land

#### Land use and land cover

There is a significant correlation between Land Use and Land Cover (LULC) and distributions of mosquito species [48]. The geography of vector abundance is a product of numerous factors including the availability and productivity of aquatic habitats, proximity of larval habitats to sugar and blood meal sources, and species-specific site preferences. LULC data were acquired from the GlobCover 2009 V2.3 product [49]. While MODIS land-use/land-cover data (MCD12Q1) offer a finer temporal resolution, it does not consistently characterize the complex mosaic of land cover in Malawi, with subtle thresholds determining land cover classifications [50]. To determine whether a class of LULC was suitable, class descriptions from the LULC data product were compared to habitat requirements of the species based on the literature review described below (Table 2).

**Table 2:** ESA GlobCover 2009 global landcover map classifications.

Class ID	Class Description	Suitable An. gambiae Land Cover
11	Post-flooding or irrigated cropland	Yes
14	Rainfed crops	Yes
20	Mosaic cropland (50–70%) / vegetation (grassland, shrubland, forest) (20–50%)	Yes
30	Mosaic vegetation (grassland, shrubland, forest) (50–70%) / cropland (20–50%)	Yes
40	Closed to open (>15%) broadleaved evergreen and/or semi-deciduous forest (>5m)	No
50	Closed (>40%) broadleaved deciduous forest (>5m)	No
60	Open (15–40%) broadleaved deciduous forest (>5m)	No
70	Closed (>40%) needleleaved evergreen forest (>5m)	No
90	Open (15–40%) needleleaved deciduous or evergreen forest (>5m)	No
100	Closed to open (>15%) mixed broadleaved and needleleaved forest (>5m)	No
110	Mosaic forest-shrubland (50–70%) / grassland (20–50%)	Yes
120	Mosaic grassland (50–70%) / forest-shrubland (20–50%)	Yes
130	Closed to open (>15%) shrubland (<5m)	Yes
140	Closed to open (>15%) grassland	Yes
150	Sparse (>15%) vegetation (woody vegetation, shrubs, grassland)	Yes
160	Closed (>40%) broadleaved forest regularly flooded—Fresh water	No
170	Closed (>40%) broadleaved semi-deciduous and/or evergreen forest regularly flooded—saline water	No
180	Closed to open (>15%) vegetation (grassland, shrubland, woody vegetation) on regularly flooded or waterlogged soil—fresh, brackish or saline water	Yes
190	Artificial surfaces and associated areas (urban areas >50%) GLOBCOVER 2009	No
200	Bare areas	No
210	Water bodies	No
220	Permanent snow or ice	No
230	Unclassified	No

*An*. *gambiae* s.s. larval habitats are characterized as open sunlit pools [51] that are not completely shadowed by vegetation [25]. Habitats occupied by larvae and productive for pupae include soil burrow pits, drainage channels and ditches within planted fields, aggregations of cattle hoof prints, tire ruts and road puddles, and other similar ground water habitats created by humans and often associated with agriculture, deforested and cultivated highland swamps [27, 52, 53]. Further, *An*. *gambiae* habitats are often associated with human activity including rice cultivation, wheel ruts, and cropping systems that facilitate water pooling [25]. To that end, GlobCover Class ID’s 11, 14, 20, and 30 (Table 2) were set to suitable for model construction. The environmental niche of the M- and S-forms of *An*. *gambiae* s.s. (now known as *An*. *coluzzii* and *An*. *gambiae* s.s. respectively) were assessed by Simard et al. [54] in Cameroon. Results showed that habitat suitability for S-form mosquitoes included dry savannah, areas of higher evapotranspiration and lower water vapor pressure, and spaces highly degraded by human activity; S-form’s avoided evergreen forest, preferring dry savanna and deciduous forest [54]. M-form mosquitoes preferred forested areas, greater sunlight exposure, higher water vapor pressure, and lower temperatures and evapotranspiration. To account for these preferences in landcover, GlobCover Class ID’s 110, 120,130, 140, and 180 were set to suitable.

#### NDVI

The Normalized Difference Vegetation Index (NDVI) is a measure of vegetation presence, health, and canopy leaf area index [55] and is calculated as a ratio of the Red and Near-infrared (NIR) spectral bands:

$$NDVI=\frac{\left(NIR-Red\right)}{\left(NIR+Red\right)}$$

Higher values of NDVI are associated with healthier vegetation or plants with substantial leaf biomass, whereas lower values typically signal poor vegetative health, or little to no vegetation present. The NDVI measure is used here as an identifier for suitable land areas for larval breeding sites and mosquito development. Vegetation has several functions during mosquito’s life history including providing necessary plant sugars for energy and nutrition [56]. Vegetated areas also provide natural resting sites, particularly for mosquitoes that preferentially feed outdoors. During resting-periods, shade provided by vegetated cover may inhibit excess water loss, reducing mosquito’s risk of dehydration and desiccation [57].

Vegetative cover is an important factor in the distribution of larval habitats. For example, in contrast to *An*. *gambiae* s.s.*’* preference for dominantly sunlit pools, *Anopheles flavirostris* is characterized as ‘shade-loving’ for its propensity to breed in pools that are partially shaded [58]. Further, shade from overhanging plants may reduce the risk of predation on mosquito larvae and provides protection from surface disturbance [59]. Studies on the relationship between mosquito species presence and NDVI are prevalent among the literature (e.g., [60–62]). Notably, Kelly-Hope, Hemingway, & McKenzie [63] found that mean NDVI values measured in three mosquito species habitats (*An*. *gambiae* s.s., *An*. *arabiensis*, and *An*. *funestus*) ranged from 0.46–0.52 in Kenya. Findings showed mean NDVI was significantly correlated with each of the three species; *An*. *funestus* was positively correlated with NDVI while *An*. *gambiae* s.s. and *An*. *arabiensis* were negatively correlated [63]. Similarly, other studies have established a relationship between NDVI and the presence of infectious disease vectors such as the tsetse fly, attributing the relationship to available moisture [64].

Beyond an association with mosquito distributions, there is a well-established relationship between NDVI and malaria (e.g., [65–67]). Hay, Snow, & Rogers [66] demonstrated that malaria infection was associated with a minimum NDVI threshold of 0.3–0.4 at three sites in Kenya. These findings were corroborated by the work of Sewe, Ahlm, & Rocklöv [67] who found that malaria mortality incidences were correlated with NDVI values in the 0.3–0.4 range, but also demonstrated that larval mortality decreased as NDVI increased. Considering the findings from these studies, a value of ≥0.35 was adopted herein to define suitable areas and the LULC data described above was used to filter out areas with the greatest canopy cover and dense vegetation (e.g., evergreen and deciduous forests). NDVI data were acquired from the MODIS Terra Vegetation Indices 16-day Global archive (MOD13Q1 V006) at a spatial resolution of 250-m.

### Water

#### Precipitation

Water is critical for mosquito larvae development and survival; mosquito distributions should covary with precipitation. To estimate inundated, or likely to become inundated areas that would support breeding, annual average precipitation was calculated from the Climate Hazards Group InfraRed Precipitation with Station Data (CHIRPS Version 2.0) product at a resolution of 0.05 arc degrees (~5.5km) [68]. Mosquito eggs are laid either on or in water, or in areas likely to pool; only a film of water is necessary to support mosquito development through the larval and pupal stages [56]. Estimating an accurate precipitation range that provides adequate water resources for breeding pool formation to support larval development is an under researched area of inquiry and may require complex hydrological modeling. Lindsay et al. [69] examined the relationship between climate variables (including precipitation) and the geographic ranges of *An*. *gambiae* s.s. and *An*. *arabiensis* throughout Africa. Results showed that total annual precipitation necessary for *An*. *gambiae* s.s. ranged between 330–3224mm [69].

Rainy season analysis showed that the 330 mm minimum precipitation threshold rendered virtually all of Malawi suitable for *An*. *gambiae* s.s. habitat during the rainy season; everywhere in Malawi is justifiably suitable at a suboptimal level during this season. In order to locate the most suitable areas, precipitation amount was parsed by quartiles. The top quartile (Q4) was adopted to locate areas that are most prone to water inundation, while still abiding by the annual maximum threshold described by Lindsay et al. [69] (3224mm). The production of larval habitats should expand and proliferate during periods of seasonal rainfall and accumulated precipitation.

#### Flow accumulation

Flow accumulation (FA) assists in identifying areas prone to ponding based upon movement of sheet water and channeled drainage characteristics of the landscape. These data assist in water flow, water channels, dambo catchment areas, and are necessary for calculating other hydrologic indices, such as the Topographic Wetness Index (TWI), that have been used in previous mosquito distribution models (e.g., [12, 70]). In Kenya, McCann et al. [12] showed that TWI along with distance to nearest stream were the two most important environmental variables for predicting *An*. *gambiae* s.l. larval habitats. FA and TWI differ only slightly; TWI is calculated as a function of FA and slope of a landscape. To delineate probable breeding habitat as a function of FA, the World Wildlife Fund HydroSHEDS Flow Accumulation mapping product at a spatial resolution of 15 arc-seconds (~500-m) was used. Given the 500-m spatial resolution of this product, areas in close proximity to the flow accumulation areas become inherently characterized as suitable, which was the desired effect. The HydroSHEDS Flow Accumulation product is based on elevation data derived from NASA’s Shuttle Radar Topography Mission (SRTM) [71]. To calculate FA, Digital Elevation Models are used to determine the natural drainage from a given pixel to adjacent, downslope pixels. Based on flow direction, the accumulated flow to each pixel is calculated [72]. Suitable areas (i.e., streams, water channels, and dambo areas) were determined by setting a threshold that corresponded with visual interpretations of satellite images in Malawi.

#### Water resources

A water resources layer was used to capture areas along the margins of permanent and large water systems (e.g., rivers, streams, and lakes) that are likely to pool and support vector breeding. Water resources were identified using the JRC Global Surface Water Bodies Mapping Layer, v1.0 data product [73]. These data were generated from a composite of >3 million Landsat 5/7/8 scenes acquired between March 16, 1984 and October 10, 2015 at a spatial resolution of 30-meters [73]. Water bodies were buffered by 250-m to approximate water-rich soils within proximity to permanent water areas. A 250-m buffer was selected to match the spatial resolution of the flow accumulation layer and maintain consistency in characterizing water prone areas. Primary water bodies and stream/river channels were subsequently masked to remove areas where larvae habitat is unsupported due to regular disturbance (e.g., water flow and waves).

## Results

### Seasonal Anopheles niche and targeting vulnerable areas

**Fig 3:**
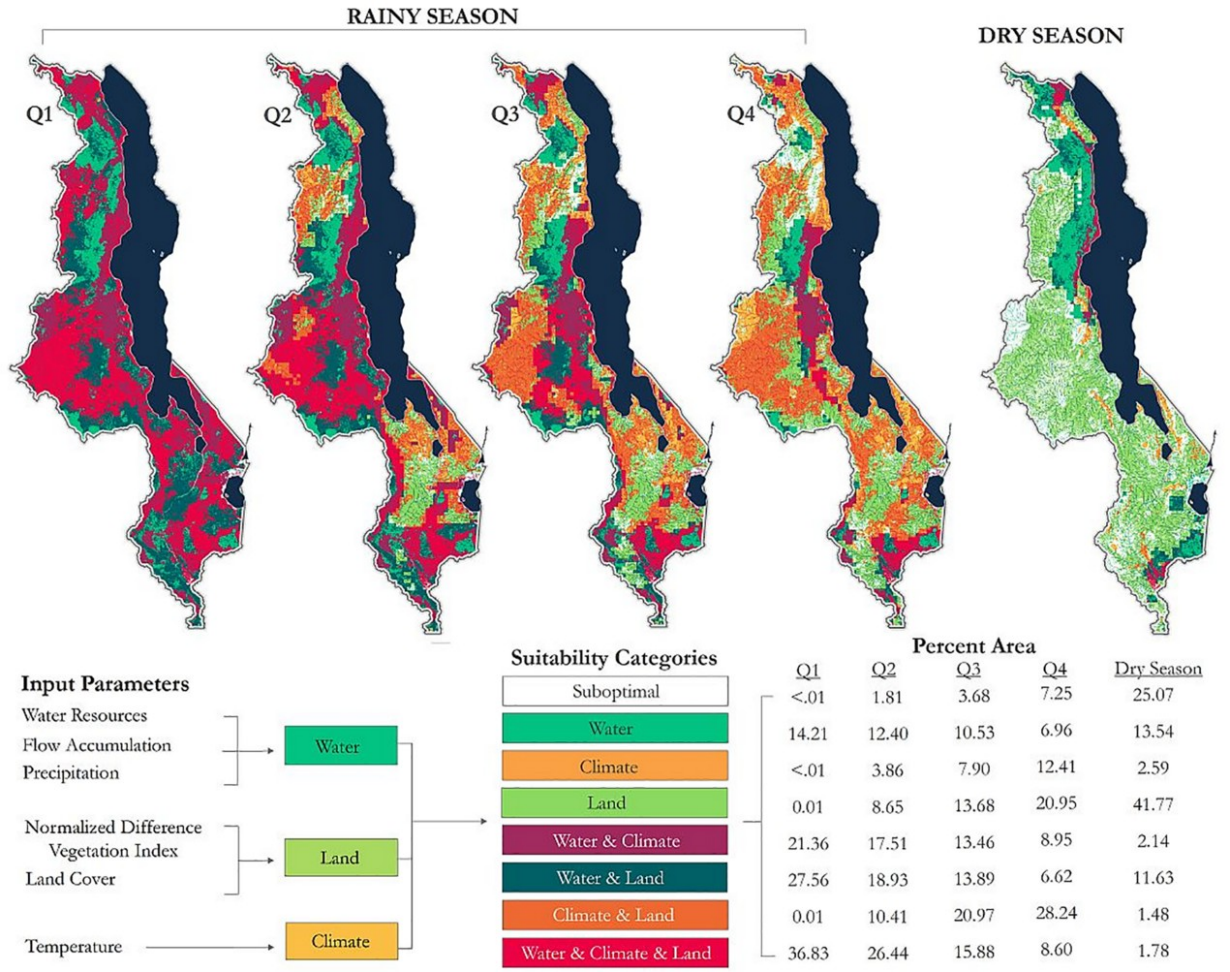
Spatio-temporal variations in habitat suitability in Malawi. Rainy season results are shown for each quartile of precipitation; Q4 is indicative of area most likely to become inundated.

Rainfall in Malawi follows a distinct unimodal pattern; average rainfall is 1305mm during the rainy season and 342mm in the dry season [74]. Mosquito abundance varies between rainy and dry seasons, due in part to the availability and quality of breeding sites [75]. Rainy Season (December–March) and Dry Season (April–November) maps are presented to elucidate the spatio-temporal variations in habitat suitability driven by seasonality. Rainy season results are presented for each quartile of precipitation; Quartile four (Q4) identifies areas most likely to become inundated (Fig 3). Mapping a range of potential inundation areas from precipitation provides useful information for developing spatially and temporally-targeted management strategies that are resource efficient and can address areas most vulnerable to mosquito production and malaria transmission. Using all four precipitation quartiles for characterizing suitability depicts the maximum extent of *An*. *gambiae* s.s. fundamental niche; however, parsing suitability classifications in this manner can benefit government and other aid stakeholders in scenarios where available resources cannot meet the complete demand and allocation determinations needed to be made based on risk. One inherent challenge associated with a quartile approach is the influence of the Modifiable Areal Unit Problem (MAUP) [76]: Different areal units will contain different ranges of precipitation, resulting in characterizations unique to each area. However, the generalizability of this method manages MAUP by allowing classifications to vary across administrative levels [77]; a country-scale classification will benefit national-scale decisions, while a district-scale classification will benefit policymakers at the district level. An alternative approach would be to use universal thresholds to define areas of water inundation, but this approach may also require complex hydrological modeling and accurate soil characteristics data.

According to Q4 results, 7.25% of Malawi exhibits suitable water conditions (water only) for *Anopheles gambiae* s.s., approximately 16% for water plus another factor, and 8.60% is maximally suitable, meeting suitability thresholds for water presence, terrain characteristics, and climatic conditions. Overall, approximately 44% of Malawi is highly prone to water inundation during the rainy season, though a larger proportion of the country is water-suitable (refer to quartiles 1–3 in Fig 3). Nearly 21% of Malawi is suitable for breeding based on land characteristics alone and 28.24% is suitable according to climate and land characteristics. Only 6.14% of the total land area is suboptimal, predominately located in northern Malawi near Mzuzu and characterized by broadleaved deciduous forest; these areas only meet suitability criteria for NDVI. Dry season results show 25.07% of the total land area is suboptimal or unsuitable. Approximately 42% of Malawi is suitable based on land characteristics alone during the dry season, and 13.11% is suitable based on land plus another factor. Less than 2% meets suitability criteria for climate, water, and land criteria (Fig 3).

### Decadal aggregations: Anopheles niche by month

**Fig 4:**
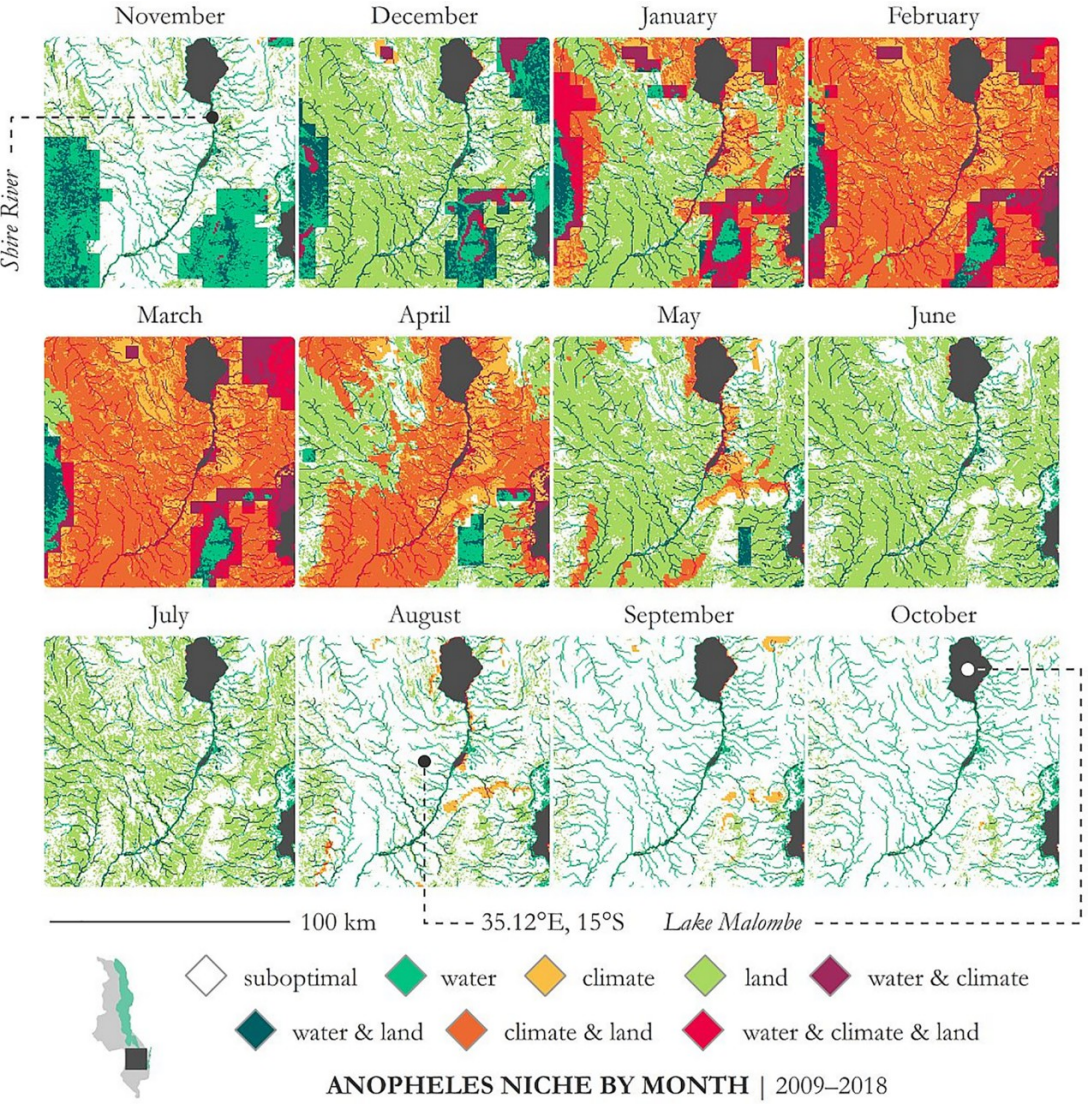
*An*. *gambiae* s. s. suitability for each month averaged across 2009–2018 in southeastern Malawi near Lake Malombeand the Shire River. Suitable precipitation is demarcated using quartile 4, i.e., areas most likely to become inundated—refer to Fig 3.

Mosquito habitat and environmental niche are inextricably linked. While the biophysical constraints that permit mosquito persistence may be definitionally static, seasonal variations in climate impact the spatial arrangement of environmental niche across temporal scales. Fig 4 demonstrates two temporal scales of observation—the rainy and dry seasons across 2017 and 2018—and Fig 4 illustrates monthly *An*. *gambiae* s.s. niche across a decade (2009–2018). Biophysical conditions for each month are averaged across the 10 years, then parsed into suitability categories using the thresholds from Table 1. Suitable precipitation is demarcated here using quartile 4. This series of maps tracks what one would expect with niche contracting during the dry season and expanding during the rainy season, with maximum suitability occurring during the peak of Malawi’s crop production season (January, February, and March). The area proportion that each suitability category occupies during each month is described in Table 3. In February and March, approximately 13.24% and 12.9%, respectively, of the area under observation contain conditions maximally suitable for *An*. *gambiae* s.s. (i.e., climate and land are both suitable and water inundation likelihood is highest). In February, only 0.03% of the area is suboptimal; 92.04% is optimal for climate and 68.99% for land. September and October are the least suitable months, with suboptimality characterizing approximately 83.89% and 85.06% of the area, respectively.

**Table 3:** Land area percentage of each category within the geographic bounds of Fig 4 for each month.

	Nov	Dec	Jan	Feb	Mar	Apr	May	Jun	Jul	Aug	Sep	Oct
**S**	57.56	18.38	7.35	0.03	0.15	10.07	23.3	27.88	44.35	75.29	83.89	85.06
**W**	26.57	11.87	4.31	2.85	3.28	3.17	4.03	4.77	5.81	8.86	10.79	11.08
**C**	0.07	0.49	9.21	16.18	16.33	14.15	3.09	0.03	0	1.43	1.26	0.06
**L**	6.83	48.38	37.79	1.2	1.92	23.08	56.19	60.2	43.79	11.15	2.95	3.02
**WC**	0.21	3.87	10.14	11.96	11.26	3.64	0.72	0.06	0.02	0.27	0.19	0.04
**WL**	8.76	16.4	7.87	3.89	3.49	3.28	7.26	7.01	6.02	2.66	0.84	0.73
**CL**	0	0.04	14.26	50.66	50.67	37.88	4.83	0.04	0	0.28	0.06	0.01
**WCL**	0.01	0.57	9.07	13.24	12.9	4.72	0.57	0.02	0	0.07	0.03	0

S = suboptimal, W = water, C = climate, L = land, WC = water & climate, WL = water & land, CL = climate & land, WCL = water & climate & land.

## Discussion

### Niche expansion, contraction, and suitability corridors

This study is the first to present modeled *An*. *gambiae* s.s. habitat suitability for Malawi based solely on environmental factors wherein clear spatio-temporal distinctions can be made of the drivers of suitability during both the rainy and dry seasons. Understanding the spatial heterogeneity of suitability for malaria mosquitoes is an important component for malaria prevention efforts, particularly for vector control-mediated reduction strategies. Though this study focused specifically on modeling *An*. *gambiae* s.s., results may elucidate areas of *An*. *arabiensis* suitability, as well. *An*. *arabiensis* and *An*. *gambiae s*.*s*. are sympatric [78]—their distributions often overlap and both species are primary malaria transmitting mosquito species in Malawi [79].

There are marked seasonal differences in suitability for *An*. *gambiae* s.s. in Malawi. Higher temperatures and the scarcity of water bodies for oviposition substantially decrease the likelihood of breeding during the dry season; however, some areas maintain suitability across seasons. Of particular note is a lengthy corridor extending along the northern and central regions of Lake Malawi that persists during the dry season, as well as suitable patches in the southern and central regions (Fig 3). One unsurprising hypothesis emerging from this research is that malaria vector mosquitoes may be concentrated in suitable dry season patches, expanding outward as seasonal climate permits (i.e., periods of rain, vegetation abundance, and hospitable temperatures). These patches may in part be related to the dambo hydrologic features of much of the Malawi landscape, which reflects the dynamic nature of the local water shed in undulating landscapes of shallow relief and relatively high water table in the dambo “valleys” between higher ground [80, 81]. This structure is discernable in Fig 3. If seasonal patterns of expanding and contracting habitat exist and are tractable, then larval source management, either through habitat modification and manipulation or larviciding (See: [82]), may benefit from targeting the more circumscribed albeit suitable dry season patches where mosquito populations are isolated and concentrated over smaller areas than during the wet season.

As for the lengthy corridor of suitability following alongside the lake during the dry season, this may be one of the dominant hubs for malaria mosquitoes to persist in the absence of seasonal rains, maintaining population sizes that can expand outward to much of Malawi during the rainy season. These patches and corridors are consistent with the findings of Kazembe et al. [83] and Mathanga et al. [34], who report that areas concentrated along the Lake Malawi lakeshore and central plains experience the highest risk of malaria infection. In this regard, environmental modifications including site-clearing and filling, drainage, or the development of ecological barriers [84] may be viable strategies that assist in disrupting long corridors and patches. Irrigated systems increase the overall suitable area for malaria vector mosquitoes [85]; however, relatively unconsidered is the potential for irrigated agriculture systems to create corridors between suitable habitats that otherwise would have remained isolated.

The absence of rainfall during the dry season limits breeding opportunity for *An*. *gambiae* s.s. However, previous research in Malawi’s Lower Shire valley has demonstrated that mosquito abundance in irrigated sites was not significantly affected by the dry season [86]. Irrigation provides consistent water sources for mosquito breeding irrespective of seasonal rainfall. This is relevant given continued expansion of irrigated agriculture across Malawi driven by food insecurity concerns (see: [87]). Changes to the geography of irrigated sites will influence malaria vector distributions. To capture these sites, the ‘Post-flooding or irrigated cropland’ class in the GlobCover 2009 product was demarcated as suitable. However, approximately 80% of Malawians are small-holder farmers [88] who cultivate relatively small parcels of land. Informally irrigated lands do not have formal planning, design, or engineered works [89] and are recorded by Malawi’s Department of Irrigation; recorded sites are as small as 0.3-hectares [89]. Given the 300-m spatial resolution of GlobCover, it is reasonable to assume that not all irrigated cropland is captured by the GlobCover product. Future work should seek to include geospatial data of irrigated sites across geographical areas of interest. These data may better characterize suitable areas for mosquito species not captured by existing LULC datasets.

### *Anopheles* fundamental niche in Malawi

Modeled results provide fine-resolution explicit information on the drivers of *An*. *gambiae* s.s. suitability across Malawi where each pixel represents a 30x30-meter area (the finest spatial resolution of all data inputs). Arguably, the largest source of uncertainty for this particular model stems from the spatial and temporal resolutions of data used for model construction (e.g., CHIRPS and MODIS LST). One limitation in measuring temperatures with remote sensing during the rainy season is the abundance of cloud cover that prevents frequent temporal measurements; however, these results are still an improvement over existing global models that are spatially and temporally discontinuous. At coarse spatial resolutions, it is assumed that conditions within each pixel are homogeneous. Although it is possible that suitable patches of *An*. *gambiae* s.s. habitat may be finer than the input spatial and temporal resolutions (e.g. Land Cover at 300-m), there are fragments of landscape containing aggregations of larval habitats at this scale (See Fig 1 in [27]; Fig 6B in [90]). Finer spatial and temporal resolution products will be needed moving forward to adequately capture local scale climate and biophysical conditions. Such products will enable advancements in modeling not only suitable habitat, but vector movement, identifying corridors, and locating smaller landscape depressions that are susceptible to pooling. Further, the ability to reliably detect the presence of human and animal populations will improve future models given the importance of blood-feeding to mosquito persistence. Despite current limitations, the flexibility of the heuristic presented here allows for easy integration as new and improved products are ingested into the Google Earth Engine data repository.

An important consideration of this study is that results elucidate geographical spaces wherein environmental conditions permit the species to exist. As such, models are constructed irrespective of species presence data. To include species presence data in our model is a movement from modeling fundamental, to realized niche, implicitly introducing the influence of confounding variables such as predation and competitive exclusion. A limitation of this model, and any remote sensing-based model, is that the outputs generated are susceptible to inaccuracy contained within each of the input products, potentially compounding as more variables are added [91]. An example native to the CHIRPS data product was demonstrated in a study conducted in Mozambique by Toté et al. [92], which showed that modeled rainfall from CHIRPS outperformed both the FEWS NET and TARCAT rainfall estimation models; however, CHIRPS tended to overestimate the frequency of rainfall events. A recent study by Duan et al. [93] evaluated the MOD112A2 LST data product with *in situ* measurements, finding that inaccurate estimates can be attributed in part to landcover misclassifications. Uncertainty issues inherent in each input variable affects the validity of the model presented here; however, this heuristic is unique in that it was designed to accept new ‘best available’ data products as they emerge. In addition, modeled outputs are a direct reflection of parameter thresholds whose values are taken from the literature. We assume the accuracy of our product in so far as the parameter thresholds used to construct the model are correct. It is prudent to note that while our models demonstrate fundamental niche of species, species realized niche may differ [14].

### Comparability with Malaria Atlas Project

Our approach and outputs are complementary to Malaria Atlas Project (MAP) products, though are distinct in several ways. Vector occurrence data from MAP ([5, 13, 24] are SDMs constructed from species occurrence, background data (e.g., previous study records on sibling species [13]), and environmental data to predict species distributions. Our work is not an SDM, rather a model that elucidates the biophysical drivers of a species fundamental niche accounting for the full range of values for environmental covariates the species has been shown to survive within. This is an important distinction in approaches between our model and MAP’s data. In traditional SDMs species presence and background and/or absence data are integral to model construction; environmental covariate values for those sites where presence, background, or absence data are recorded will influence model results. The inclusion of background presence/absence data in SDMs are a means at limiting environmental bias (stemming in part from sample selection bias), however these data may still not fully reflect the complete fundamental niche a species may survive within. It is prudent to note that fundamental niche is not synonymous with species presence. Realized species distributions are the result of a complex interplay of environmental and anthropogenic factors that influence the survival of species [16]. The results of this study indicate where environmental characteristics would allow *An*. *gambiae* s.s. to exist; it should not be taken as a perfect representation of *An*. *gambiae* s.s. distribution or presence. However, it is assumed that where more environmental variables converge on suitability, there is a higher likelihood of species presence.

An added marked difference in our heuristic compared to MAP data products is the user’s ability to adjust parameter thresholds based on unique knowledge of target species and study area. Variable thresholds that may be modified are temperature ranges, minimum NDVI, precipitation ranges, LULC, temporal ranges, and flow accumulation area delineation. Start and end year, or range of months (for seasonal inquiries), provides an opportunity for examining fine-resolution, spatio-temporal information on the drivers of suitability over time within the user-defined geography. While data from MAP provide critical information on which environmental parameters are significant predictors of species presence, the functionality of our model allows users to investigate not only the geography of suitable areas, but what variables, or combinations of variables encourage or restrict the likelihood of the species inhabitation across both space and time. Finally, there is a stark difference in resolution of outputs: 30-m and 5-km for our model and MAP data, respectively.

**Fig 5:**
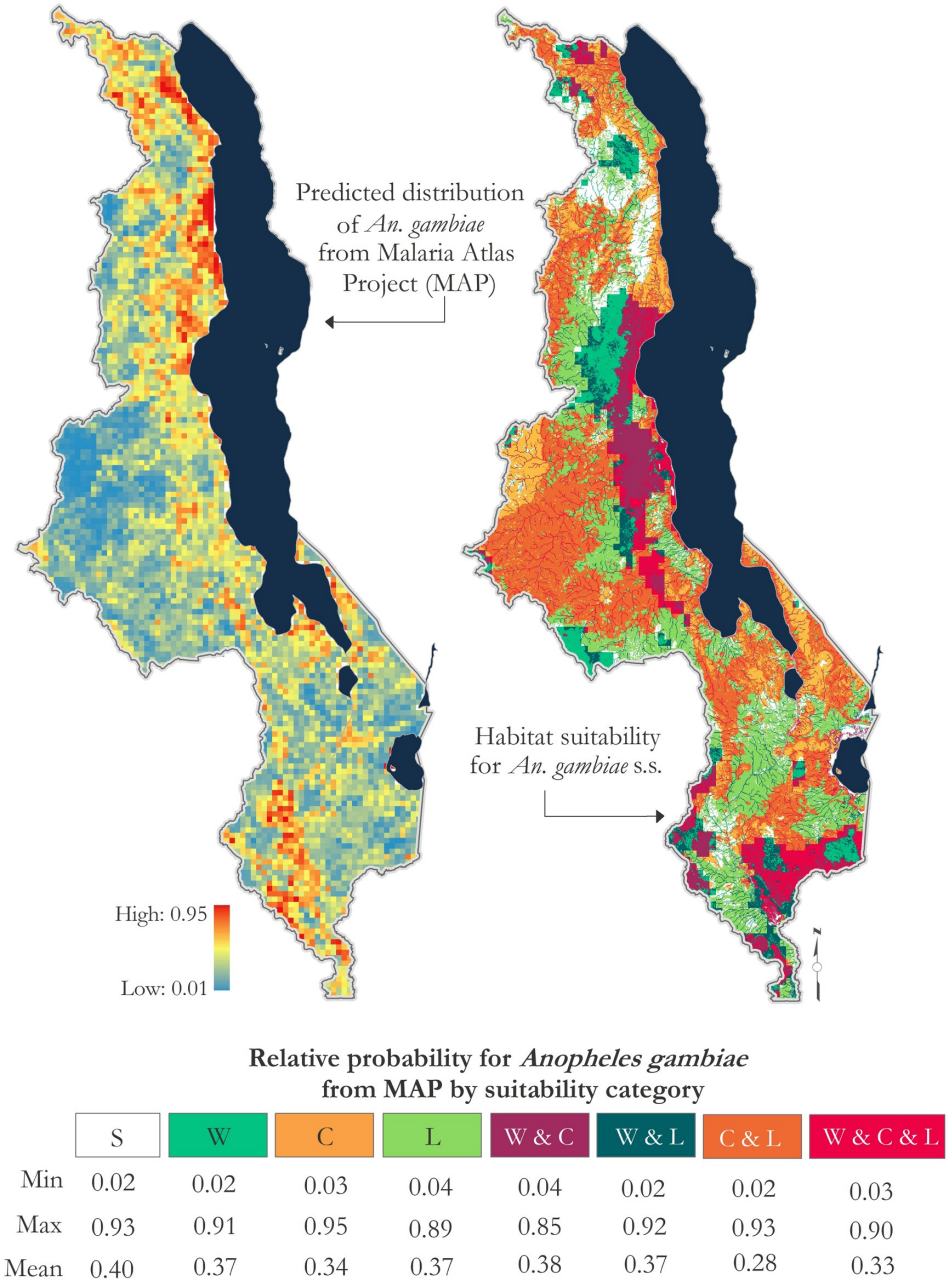
Relative probability for *Anopheles gambiae* from Malaria Atlas Project (MAP)—Vector Occurrence (Current) data [13] by suitability category.

Despite the aforementioned marked differences between our model and predicted MAP distributions, we compared our modeled rainy season Q4 outputs for *An*. *gambiae* s.s. in Malawi to data from the MAP’s current data product for *An*. *gambiae Giles*, *1902* in Africa [13] by calculating percent relative probability of vector occurrence within the area delineated by each suitability category (Water, Climate, Land, Water & Climate, Water & Land, Land & Climate, Water & Climate & Land). Modeled Q4 outputs reflect areas most likely to be inundated and it was assumed here that predicted vector distributions from MAP and Q4 areas are conceptually analogous. Results showed little variation in the minimum, maximum, and mean relative probability values of vector occurrence with each suitability category area (min range: 0.02–0.04; max range: 0.85–0.95; mean range: 0.28–0.40) (Fig 5). Mean values are highest in areas classified as suboptimal in our model (0.40).

The spatial resolution of environmental predictor variables used in model construction here, as with MAP, can impact the resolution or scale of the conclusions drawn. The spatial resolution of data layers used to model species distribution for MAP data is 5-km. While precipitation data used in our model are ~5.5-km resolution, the remainder of input data spatial resolutions are much finer. As a result, significantly more microscale variation is elucidated in our model. An added consideration is temporal resolution of input data layers. Data layers used in model construction for MAP data are annual data and may only reflect the relative probability of occurrence for the modeled species at one point in the year. Depending on the timing and frequency of input species presence/background data, results may be biased to reflect environmental conditions associated only with collection times. The Q4 data from our model used in this analysis is representative of rainy season months (December–March) in Malawi. Further, it is important to note the difference in analysis scale of our model and the MAP data. MAP data for Malawi used in this analysis were subset from Wiebe et al.’s [13] 2017 predicted geographical distributions for *An*. *gambiae*, constructed at the African continental scale. Malawi, like much of sub-Saharan Africa, experiences strong seasonal variations in climate that differ from other ecological zones across Africa and affect the spatio-temporal distribution of mosquito abundance.

## Conclusions

Modeling environmental suitability based on the physiological requirements of species facilitates research and vector control strategies in areas where species data may be limited or absent. Defining geographical distributions of malaria-vector species is an integral part of malaria control programs, yet so too is understanding what factors contribute to higher or lower relative probabilities of occurrence. Malaria control programs in Africa predominately aim to reduce human-vector contact through the use of long-lasting insecticidal nets and indoor residual spraying [24, 94]. Yet, Sinka et al. [25] highlight that these technologies are often implemented without considering species distributions, composition, or behavior. Elucidating the environmental drivers of suitability for malaria vectors provides an opportunity for a more comprehensive approach to malaria control that includes not only modeled species distributions, but also the underlying drivers of suitability for a more effective approach to environmental management.
